# Communication challenges of a tobacco addictiveness reduction policy

**DOI:** 10.18332/tid/134747

**Published:** 2021-05-12

**Authors:** Stella Bialous, Becky Freeman

**Affiliations:** 1 Social and Behavioral Sciences Department, School of Nursing, University of California, San Francisco, United States; 2 Prevention Research Collaboration, Charles Perkins Centre, School of Public Health, Faculty of Medicine and Health, University of Sydney, Sydney, Australia

**Keywords:** nicotine, addiction, comprehensive tobacco control, public health communication

While nicotine has long been identified as the primary addictive substance in tobacco products^1^, globally there has been minimal policy action to regulate nicotine levels within tobacco products. Current maximum limits on nicotine content in tobacco products have yet to result in any public health gains, as nicotine is still maintained at an addictive level^2^. Initial ideas for a nicotine reduction strategy involved gradually reducing the allowable level of nicotine in cigarettes to non-addicting levels, both as a way to prevent youth from becoming addicted and to help existing smokers to quit^3^. A growing body of small experimental studies suggests that removing nicotine from cigarettes may assist smokers to successfully quit, but no large-scale interventions, under real-world conditions, have yet been conducted^4^. Nonetheless, there is some interest in exploring possible policy solutions to reducing the addictiveness of tobacco products, especially given the current proliferation of novel and alternative nicotine and tobacco products.

In 2016, at the seventh session of the Conference of the Parties of the WHO Framework Convention on Tobacco Control (FCTC), Parties decided to hold a meeting to explore the positive and negative aspects of tobacco addictiveness reduction policies^5^. One of the key issues identified as part of this meeting was how such a policy, if implemented, could be effectively and proactively communicated to the public. This echoes a 2015 recommendation from the WHO Study Group on Tobacco Product Regulation (TobReg) that an ‘immediate reduction in nicotine, preceded by health communication strategies and public education’ be pursued as the most promising way of reducing the addictive potential of tobacco products^2^.

TobReg defined a policy to reduce the addictiveness of tobacco by ‘setting a maximum allowable limit on the nicotine content of all cigarettes and potentially other forms of tobacco (both combusted and non-combusted) that are available for sale, with the intention of minimizing the development and/or maintenance of nicotine addiction’^2^. Currently, there is a lack of consensus as to whether a tobacco addictiveness reduction policy would reduce tobacco use. Some of the assumed benefits of such a policy include making it easier for smokers to quit using tobacco and encouraging smokers to use alternative forms of nicotine delivery, including medicinal nicotine replacement products. For non-tobacco users that experiment with tobacco use, such a policy could make it less likely that they progress to regular tobacco use. However, it is also possible that smokers will readily obtain tobacco products containing nicotine through illicit or cross-border channels, and that non-users may mistakenly believe that tobacco products are now ‘safer’ to use if they no longer contain nicotine^6^.

The aim of our commentary is to provide a preliminary exploration of the communication challenges if a policy to reduce the addictiveness of tobacco products were to be adopted. The complex communication needs suggest substantial planning and resources would be required to help ensure the desired positive health impact. The US Food and Drug Administration (FDA) had considered pursuing such a policy^7^, but as of December 2020 this was not an active policy priority. While some nations currently regulate the nicotine concentration in electronic cigarettes and vaping devices for safety reasons^8^, there is not yet a countrywide experience of tobacco addictiveness reduction policy implementation. Unlike clinical approaches to smoking cessation that prove difficult to successfully implement at a population level^9^, a policy that reduced the addictive potential of all tobacco products would reach all tobacco users. Below, we elaborate on the key challenges that need addressing within the communications strategy, these include: misconceptions and influences on beliefs about nicotine, gaps in healthcare professional knowledge, and delivering comprehensive public education.

## Misconceptions and influences on beliefs about nicotine

Communication surrounding nicotine and addiction has been heavily influenced by the tobacco industry, which until a decade ago denied that nicotine was addictive^10^. It now serves tobacco industry purposes to position nicotine as the sole driver of tobacco addiction, as it allows the industry to ignore the broader social and environmental factors that perpetuate addiction, and reposition itself as a reformed company that manufactures safe nicotine products^10^. There is a significant risk that the tobacco industry will attempt to control or manipulate any communication strategy around a reduced addiction policy. This type of risk has already been borne out, with Philip Morris immediately misrepresenting the 2020 US FDA decision that its heated tobacco product, IQOS, was approved as a reduced harm product^11^. The US FDA only approved IQOS as a reduced exposure product and found no significant evidence that it reduced harm or tobacco-related diseases. A communication plan must not further enable the tobacco industry to present itself as a provider of cessation tools^12^, nor assist in positioning the tobacco industry as being equal with the pharmaceutical industry^13^. There is also evidence that the industry has long anticipated that there may come a time when nicotine could be removed for tobacco products and has undertaken research into possible nicotine analogues^14^.

Consumer misperceptions about nicotine and tobacco product safety further complicate what a communication plan should actually entail^15^. While the current evidence suggests nicotine does play a role in tumor growth and spread^16^, it is a common belief that nicotine itself is carcinogenic^17^. A 2016 US survey, of a nationally representative sample of 650 adult smokers, found that around 47% of participants believed that very low nicotine content (VLNC) cigarettes were less carcinogenic, and such a belief was significantly associated with a lower likelihood of quitting^18^. A 2015 US survey, of more than 4000 adults, found that 71% agreed that the FDA should require companies to reduce the nicotine level in cigarettes^19^. It is unclear if support for reducing nicotine from cigarettes is because people believe this will make cigarettes less addictive or make them safer to use. Any communication campaign would need to not only explain why nicotine was being removed from cigarettes, but also clearly address the health risks of continuing to use tobacco products that either have no or very low levels of nicotine. Focus group research with smokers found that they believe tobacco additives increase addiction, asserting that tobacco companies add substances for the express purpose of preventing people from quitting^20^. But, not all study participants who attributed addiction to additives mentioned nicotine, it is unclear whether they were unaware of the link between nicotine and addiction, or just did not name nicotine specifically.

Additionally, it is unclear how, or if, a tobacco addictiveness reduction policy would also apply to non-cigarette tobacco products, such as cigars, oral tobacco, and waterpipes. The perceptions of harms, addiction and nicotine content associated with these non-cigarette products are not as well understood as for cigarettes^21,22^. A nationally representative household survey, conducted in fourteen different countries, found that adults living in low and middle income countries have inadequate awareness as well as false perceptions about smokeless forms of tobacco use^23^.

Similar to disparities in rates of tobacco use based on socioeconomic, educational and other social determinant factors^15^, understanding of tobacco harms and nicotine also varies based on these factors. Lower socioeconomic status is associated with lower awareness of the harms of smoking and greater misunderstanding that nicotine causes cancer^24^. A study, of tobacco use knowledge among people who are HIV positive, found that while the majority of participants correctly identified smoking as being a potential cause of smoking-related conditions, the majority of participants also misattributed nicotine as a cause of smoking-related illness^25^. Reducing health inequity is a fundamental principle of tobacco control and any policy must be assessed on its potential to further increase these inequities. While an effective communication strategy could assist in addressing these inequities, if disadvantaged tobacco users are more likely to believe that these modified tobacco products are safer, this may not only discourage smoking cessation, but also discourage use of safer nicotine replacement products.

One possible outcome of a policy to reduce the addictive potential of tobacco products is that it will also assist in aiding smokers to switch to alternative nicotine products, such as electronic cigarettes and nicotine vaping devices. If these alternative products are not also included in the policy to reduce addictiveness, they could be more attractive to tobacco users and experimenters when they no longer have to compete with traditional tobacco products. If tobacco products do not contain nicotine (or only have very low amounts), then the general idea is that these alternative nicotine products, again if they are also excluded from the policy, could become more favorable to users. The uncertainty surrounding product details, and product regulation of electronic nicotine devices, adds yet another layer for public misunderstanding. For example, a 2018 survey of young people’s perception of a pod-style vaping product, Juul, showed that 63% of those surveyed did not know that the product contained nicotine^26^. While current marketing related to these products, where available and allowed, emphasize the potential for reduced health risk^27^, there is no evidence to suggest that these products are any less addictive. Currently, concurrent use of both cigarettes and vaping devices is common^28^, and there is no robust evidence available to help understand whether removing nicotine from smoked products will decrease or increase concurrent use with vaping devices. Furthermore, these products are not universally available, with some countries banning or heavily restricting access due to valid health and safety concerns^8^. The potential impact and communication needs of a policy to reduce tobacco product addictiveness would also depend on the scope of the proposed policy and the regulatory environment of the country.

## Gaps in healthcare professional knowledge

A systematic review of what training characteristics prepare healthcare professionals (HCPs) in the delivery of care to tobacco users found that most HCPs receive limited education related to tobacco dependence treatment, including little education on nicotine addictiveness, withdrawal, and available therapies^29^. For example, Greek healthcare professionals appear to overestimate the adverse effects of nicotine, and more than 30% considered nicotine replacement therapies equally or more addictive than smoking^30^. A communications strategy would need to include a focus on educating HCPs on the potential benefits and impact of tobacco addictiveness reduction policy. HCPs are also key tobacco control policy stakeholders and would need to be involved in all stages of policy development and implementation, including a communication strategy.

## Public education

Educating the public about the harms of tobacco use through the use of both mass media campaigns and on-pack health warnings is effective in encouraging smoking cessation and preventing smoking initiation. While on the surface the messaging behind these campaigns may appear ‘simple’ – don’t smoke, quit smoking if you do – effectiveness depends upon campaign reach, intensity, duration, and the emotional appeals deployed in the campaigns^31^. As of 2018, 118 jurisdictions, covering more the 58% of the global population, require graphic-based warnings directly on tobacco packages, with a common warning being that tobacco products are addictive^32^. It is unclear if under a tobacco addictiveness reduction policy, this addiction warning would need to be removed and how this removal would effect public trust or understanding about tobacco-related harms. A successful tobacco control mass media strategy requires ongoing investment and sufficient population exposure. Much of the work to date on effective tobacco control campaigns has been focused on broadcast television, while understanding the best use of digital and social media in public health messaging is underdeveloped^33^. Any communication strategy around a tobacco addictiveness reduction policy must encompass social media as the risk of the spread of misinformation for such a policy is high. Lessons learned, from the rapid spread of false information surrounding other public health issues, may be valuable in informing a campaign^34^.

## Future research and next steps

Any communication strategy would need to convey information about country-specific products to consumers and ensure that it is not an advertising campaign that would drive non-tobacco users to use reduced addictiveness tobacco products. Monitoring media coverage of the topic of addictiveness and nicotine, and how best to influence it going forward, are necessary steps. Ongoing content analyses of news reporting and social media trends on tobacco and nicotine addiction would be practical ways of beginning this work. Future research could shed light on the reasons why people support these types of policies and how such rationale could guide the communication strategy development. Research should also explore the informational needs of politicians and policymakers to ensure that appropriate resources are allocated to communication programmes and that any policy to reduce the addictiveness of tobacco occurs within a broader framework of comprehensive tobacco control measures.

There is a large and growing body of research focusing on reducing tobacco-related harm, but research on a reduced nicotine policy has received far less attention, as most research is about communication of risk and perception of risk, not nicotine and addiction. A better understanding of perception and understanding of reduced addictiveness policy are required and would need to include consumers, health professionals, the media, politicians and policymakers. Additional research could shed light on the reasons why people support reduced addictiveness policies and how this rationale can help guide the communication strategy development. Further, the burden of tobacco use is concentrated in low and middle income countries, ensuring the views and attitudes and policy context are well understood in these settings is vital before any universal recommendation about the appropriateness of a tobacco addictiveness reduction policy could be made.

A communication strategy to support an addictiveness reduction policy would also need to educate health professionals and involve key stakeholders in all stages of policy development and implementation. Importantly, communication would ensure that the message uncouples overall tobacco harm from nicotine content and addresses the misperception that reduced addictiveness tobacco products are less carcinogenic. It would further need to ensure that the target audience is clear, the campaign is evaluated, and that investments in a communications campaign are cost effective. A communication strategy requires political and resources commitment, and an evaluation to assess impact will be a key requirement.

At this point, any recommendations are highly exploratory and based on a best-case scenario where the positive aspects of an addictiveness reduction policy far outweigh the negative aspects. If a decision is made to propose such policy, a communication strategy, planned simultaneously with policy discussions, must consider the perceptions, beliefs and informational needs of all stakeholders to support policy implementation. Careful planning, including the launch of a campaign well in advance of policy implementation, and above all, evaluation of impact will be pivotal to ensure that a communication strategy helps drive the intended outcome of the policy.

## CONCLUSIONS

There are significant obstacles in communicating clearly and effectively about tobacco use, addiction, nicotine, and health, under a potential reduced tobacco product addictiveness policy, and a risk for the message to be co-opted by the tobacco industry. Positioning harm reduction as a policy priority, in the form of alternative nicotine and tobacco products, is not only profitable for the tobacco industry, but can also divert support away from a comprehensive approach to tobacco control^35^. Nicotine reduction policies could further entrench these views if not pro-actively managed through a strategic communication plan. Proven policy drivers, that are part of a comprehensive approach – such as taxation, advertising bans, smoke-free public spaces, and graphic health warnings – that both reduce and denormalize tobacco use, must continue to be at the core of tobacco control efforts^36^. As demonstrated by the rapid and near global adoption of the WHO FCTC, policymakers across the political spectrum are supportive of comprehensive tobacco control measures, but very little is known of their knowledge or views on reduced tobacco addictiveness policies.

We are unconvinced that a reduced addictiveness policy would achieve the public health benefits envisioned, and have many reservations as to whether a communications strategy could help to address the many unintended consequences such a policy might entail. Implementation, communication and evaluation would have to be in the context of broader implementation of Articles 9 (Regulation of the contents of tobacco products) and 10 (Regulation of tobacco product disclosures) of the WHO FCTC^37^. There is growing global interest in controlling the supply of tobacco products, including prohibiting the retail sale of tobacco products^38^. This may ultimately be a more fruitful policy action in jurisdictions that have already fully implemented the suite of program and policy measures prescribed under the WHO FCTC.
